# Recruitment across two decades of NIH-funded Alzheimer’s disease clinical trials

**DOI:** 10.1186/s13195-023-01177-x

**Published:** 2023-02-02

**Authors:** Marina Ritchie, Daniel L. Gillen, Joshua D. Grill

**Affiliations:** 1 UC Irvine Institute for Memory Impairments and Neurological Disorders, University of California, Irvine, Irvine, CA 92697 USA; 2 Department of Neurobiology and Behavior, University of California, Irvine, Irvine, CA 92697 USA; 3 Department of Statistics, University of California, Irvine, Irvine, CA 92697 USA; 4 Department of Psychiatry and Human Behavior, University of California, Irvine, Irvine, CA 92697 USA

**Keywords:** Recruitment, Alzheimer’s disease, Clinical trials, Accrual

## Abstract

**Background:**

Timely accrual of a representative sample is a key factor in whether Alzheimer’s disease (AD) clinical trials successfully answer the scientific questions under study. Studies in other fields have observed that, over time, recruitment to trials has become increasingly reliant on larger numbers of sites, with declines in the average per-site recruitment rate. Here, we examined the trends in recruitment over a 20-year period of NIH-funded AD clinical trials conducted by the Alzheimer’s Disease Cooperative Study (ADCS), a temporally consistent network of sites devoted to interventional research.

**Methods:**

We performed retrospective analyses of eleven ADCS randomized clinical trials. To examine the recruitment planning, we calculated the expected number of participants to be enrolled per site for each trial. To examine the actual trial recruitment rates, we quantified the number of participants enrolled per site per month.

**Results:**

No effects of time were observed on recruitment planning or overall recruitment rates across trials. No trial achieved an overall recruitment rate greater than one subject per site per month. We observed the fastest recruitment rates in trials with no competition and the slowest in trials that overlapped in time. The highest recruitment rates were consistently seen early within trials and declined over the course of studies.

**Conclusions:**

Trial recruitment projections should plan for fewer than one participant randomized per site per month and consider the number of other AD trials being conducted concurrently.

**Supplementary Information:**

The online version contains supplementary material available at 10.1186/s13195-023-01177-x.

## Introduction

Randomized controlled clinical trials represent the final and frequently the most expensive stage in the drug development process [1, 2]. In particular, participant recruitment is a consistent challenge that can increase overall costs in neurological disease trials. Slow or inadequate recruitment can prolong trials, delay advances in care, and exhaust precious resources, drawing them away from studies of other promising therapies [3–5]. It can lead to ethical consequences such as late detection of safety signals due to lack of precision, as well as study termination without adequately addressing the scientific question of interest [6, 7].

In Alzheimer’s disease (AD) trials, unique barriers magnify enrollment challenges. AD is an age-related progressive neurodegenerative disease. Patients are frequently excluded from trials due to comorbid conditions and prohibited medications [8, 9]. AD trials also require dyadic enrollment of a participant and a study partner, exacerbating logistical challenges to participation [5]. These requirements and challenges have remained consistent, even as AD trials have evolved to enroll patients at earlier stages of the disease, such as those with mild cognitive impairment (MCI).

AD drug development has been an active area of research over the last two decades. Over this period, major advances were made in target identification, drug development, biomarkers and other diagnostic tools, and trial design [10]. We sought to assess whether similar advances in trial recruitment were made. Studies in other fields over similar periods have observed that recruitment to trials has become increasingly reliant on larger numbers of sites, suggesting a downward trend in site-level recruitment metrics [11]. To assess chronological trends in AD trial recruitment, we used data from the Alzheimer’s Disease Cooperative Study (ADCS), a stable network of primarily academic sites devoted to dementia research. Specifically, we examined whether trial planning, recruitment rates, and trial sample demographics changed over time.

## Methods

### Study design

This descriptive study examined trends over time in AD trial participant recruitment. We requested and received datasets for eleven ADCS trials initiated between 1999 and 2014 through the ADCS data sharing committee and the Alzheimer’s Therapeutic Research Institute at the University of Southern California (Table 1). Trials were 4 (1 trial), 12 (4 trials), 18 (4 trials), 24 (1 trial), or 36 months (1 trial) in duration. They included studies of the following interventions (year of initiation): donepezil /vitamin E (1999) [ClinicalTrials.gov identifier: NCT00000173] [12], nonsteroidal anti-inflammatory drugs (NSAIDs, 1999) [ClinicalTrials.gov identifier: NCT00004845] [13], simvastatin (2002) [ClinicalTrials.gov identifier: NCT00053599] [14], high-dose B vitamin supplementation (vitamin B, 2003) [ClinicalTrials.gov identifier: NCT00056225] [15], valproate (2003) [ClinicalTrials.gov identifier: NCT00071721] [16], huperzine A (2004) [ClinicalTrials.gov identifier: NCT00083590] [17], docosahexaenoic acid (DHA, 2007) [ClinicalTrials.gov identifier: NCT00440050] [18], intravenous immunoglobulin (IVIG, 2008) [ClinicalTrials.gov identifier: NCT00818662] [19], resveratrol (2012) [ClinicalTrials.gov identifier: NCT01504854] [20], intranasally administered insulin (INI, 2014) [ClinicalTrials.gov identifier: NCT01767909] [21], and the FYN kinase inhibitor AZD0530 (FYN, 2014) [ClinicalTrials.gov identifier: NCT02167256] [22]. Six trials were phase III studies, three were phase II, and two were phase II/III.

**Table 1:** Summary of key trial design features, trial planning, and actual site recruitment outcomes

Intervention	Donepezil/vitamin E	NSAIDs	Simvastatin	Vitamin B	Valproate	Huperzine A	DHA	IVIG	Resveratrol	INI	FYN
**Initiation year**	1999	1999	2002	2003	2003	2004	2007	2008	2012	2014	2014
**Trial phase**	3	2/3	3	3	3	2	3	3	2	2/3	2
**Diagnostic category**	MCI	Mild to moderate AD	Mild to moderate AD	Mild to moderate AD	Moderate AD	Mild to moderate AD	Mild to moderate AD	Mild to moderate AD	Mild to moderate AD	MCI and Mild AD	Mild AD
**Trial length (months)**	36	12	18	18	24	4	18	18	12	12	12
**Planned total sample size,** ***n***	720	320	400	400	300	210	400	385	120	240	152
**Number of sites,** ***n***	70	40	45	39	43	32	51	45	25	27	22
**Expected site recruitment, participants/site**	10.3	8.0	8.9	10.3	7.0	6.6	7.8	8.6	4.8	8.9	6.9
**Actual site recruitment, participants/site (mean, SD [range])**	11.3 ± 6.1 [0–35]	8.8 ± 3.7 [3–15]	9.0 ± 4.9 [0–23]	10.5 ± 8.4 [1–44]	7.3 ± 6.2 [0–32]	6.6 ± 4.8 [0–18]	7.9 ± 4.9 [0–23]	8.7 ± 5.5 [0–21]	4.8 ± 4.4 [0–18]	10.7± 7.9 [0–32]	7.2 ± 6.3 [1–32]
**Overall recruitment rate, screened participants/site/month**	1.47	1.19	0.41	0.57	0.31	0.25	1.21	0.54	0.65	0.40	0.73
**Overall recruitment rate, randomized participants/site/month**	0.51	0.88	0.24	0.39	0.19	0.18	0.88	0.30	0.43	0.40	0.33

Seven trials enrolled mild-to-moderate AD participants, one included only mild AD participants, one included only moderate AD participants, one included participants with MCI and mild AD, and one included only participants with MCI. The Mini-Mental State Examination (MMSE) criteria for inclusion varied according to the diagnostic population of interest. The donepezil/vitamin E trial was restricted to MMSE 24-30, the INI trial was restricted to 20–30, the FYN trial was restricted to MMSE 18–26, and the valproate trial was restricted to MMSE 12–20. Among the remaining mild-to-moderate trials, the range of lower limits was 10–16; the range of upper limits was 24–26. The trials applied similar exclusion criteria (e.g., psychiatric disorders or other conditions that may impair cognition). There were some trial-specific criteria (Additional file 1: Table S1 and Additional file 2: Table S2).

Each trial failed to demonstrate a benefit of the therapy under study based on the primary analysis of treatment versus placebo. The primary outcome of the donepezil/vitamin E trial was the rate of conversion to possible or probable AD; the valproate trial assessed time until symptoms of agitation or psychosis emerged. The resveratrol trial assessed potential biomarkers of AD including cerebrospinal fluid total tau, phosphorylated tau, amyloid beta, and volumetric magnetic resonance imaging. The primary outcome of the FYN trial was cerebral metabolic rate for glucose measured by fluorodeoxyglucose positron emission tomography. The primary outcome for the remaining trials was change in the Alzheimer’s Disease Assessment Scale cognitive subscale [23]. The DHA trial included the Clinical Dementia Rating Scale [24] as a co-primary outcome and the IVIG trial included the ADCS Activities of Daily Living Inventory [25] as a co-primary outcome.

### Statistical analyses

**Fig. 1:**
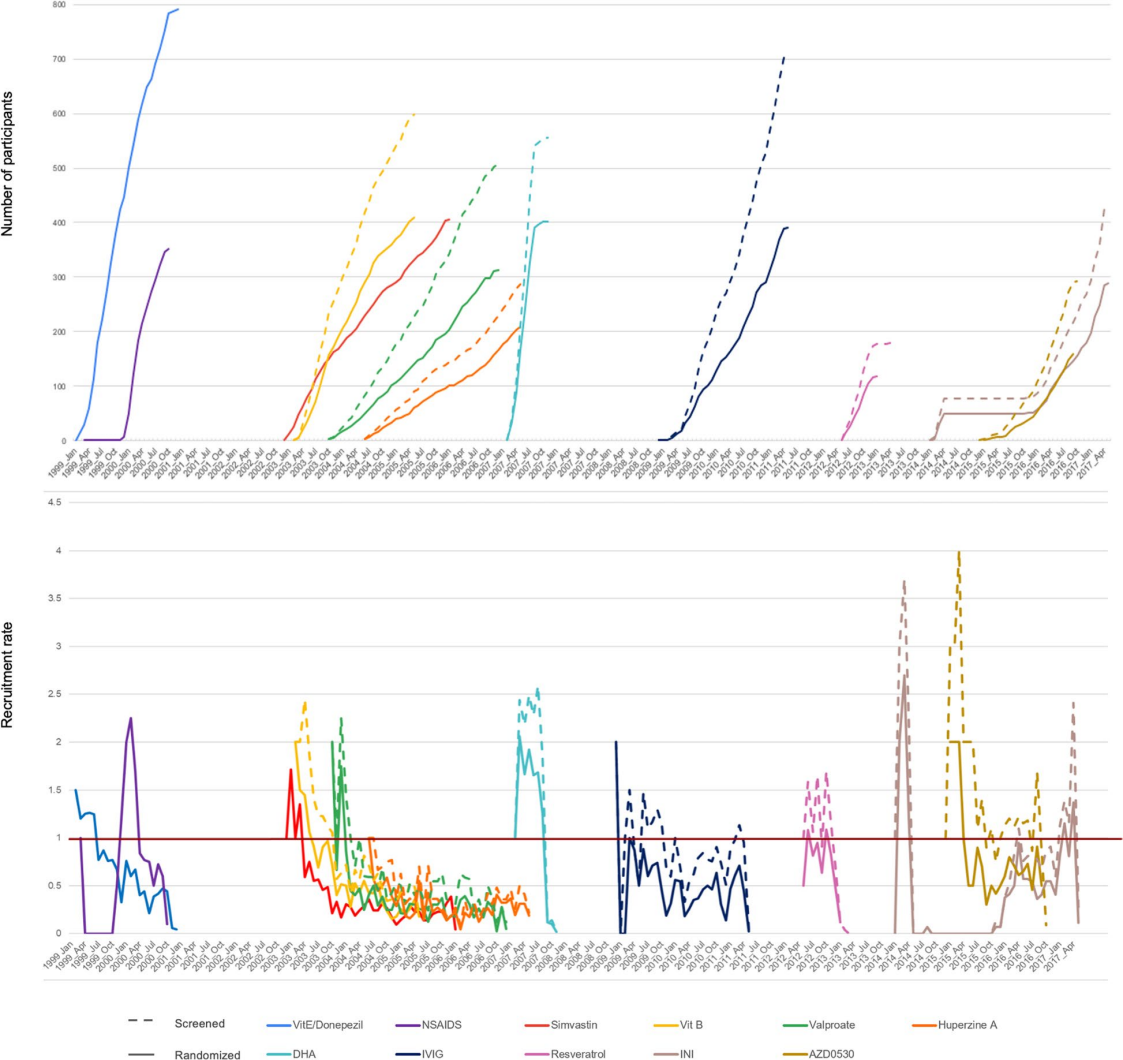
**a** Accrual patterns over time (screened and randomized participants) and **b** accrual patterns over time normalized by number of active sites at a given point within a trial. The dotted lines indicate the patterns over time for screened participants, and the solid lines indicate the patterns over time for randomized participants

We examined trial planning outcomes by calculating the average expected number of participants enrolled per site within each study. We estimated the first-order trend in expected trial recruitment per site and overall recruitment rates (screened and randomized participants/site/month) over time using ordinary least squares. The associated Wald-based confidence interval and corresponding *p*-value for a test of the null hypothesis of no change in expected recruitment over time were computed. We used the number of months indicated in the original manuscripts if reported. For the remaining trials, we used the number of months between the first and last screening date. For the INI trial, we excluded the months where recruitment was paused between the administration of two drug delivery devices. To further examine actual recruitment, we calculated overall accrual rates for a given trial and considered the number of active sites at a given point in time. We plotted accrual curves to visualize this information (Fig. 1). When possible, we considered both screening and randomization rates; screening data were unavailable for the donepezil/vitamin E, NSAIDs, and simvastatin trials. For these trials, we used the total number of screened participants published in the original manuscripts when assessing screening rates. We assessed participant demographics including age, race and ethnicity, sex, and study partner type. With two exceptions, trials collected race and ethnicity as separate variables. Using participant-level data, we re-categorized participant race and ethnicity into six mutually exclusive groups: non-Hispanic White, non-Hispanic Black or African American, non-Hispanic Asian/Pacific Islander, non-Hispanic American Indian or Alaskan Native, Hispanic (of any race), and others. When data were available, study partner types were categorized into spouse, adult child, and other categories. As an exploratory approach, we estimated the effect of time on demographic characteristics and study partner type using ordinary least squares. We used the Holm-Bonferroni method for each construct to control the family-wise type I error rate for presented hypothesis tests [26].

## Results

Expected and actual recruitment outcomes are summarized in Table 1. The expected site-level recruitment ranged from 4.8 to 10.3 participants/site. The number of planned participants per site showed a negative trend over time although the results were not statistically significant (est: − 0.13 change in participants/site/year; 95% CI: − 0.33, 0.07; *p* = 0.168). The overall screening rate ranged from 0.25 to 1.47 screened participants/site/month, and no effect of time was observed (est: − 0.02 change in rate per year; 95% CI: − 0.08,0.03; *p* = 0.320). The overall recruitment rate ranged from 0.18 to 0.88 randomized participants/site/month and demonstrated no effect of time (est:0.01 change in rate per year; 95% CI: − 0.02, 0.03; *p* = 0.685).

Accrual curves for the eleven trials are presented in Fig. 1. Trials that overlapped in time were observed to accrue more slowly than those with no competing trials (Fig. 1a). The simvastatin trial enrolled 0.24 participants/site/month followed by trials of vitamin B (0.39), valproate (0.19), and huperzine A (0.18). Within trials, the recruitment rate adjusted for the number of active sites consistently peaked early in trial conduct (first 1–4 months) and declined over the course of accrual (Fig. 1b).

Demographic characteristics of the trials’ randomized participants are summarized in Table 2. The mean participant age (est: − 0.29; 95% CI: − 0.61, 3.63; *p* = 0.0757) and proportion of females (est: − 0.36; 95% CI: − 1.14, 0.43; *p* = 0.335) did not change over time. Trials that enrolled moderate AD patients included a majority of female participants; trials of milder populations included more males. Most participants enrolled with a spouse study partner; the fewest non-spouse study partners enrolled in the most recent trials. There was an increase in the proportion of participants enrolling with spousal study partners (est: 0.95; 95% CI: 0.12, 1.77; *p* = 0.0297) over time, although the results were not statistically significant after adjusting for multiple comparisons. There was no apparent effect of time on the racial and ethnic diversity of trial samples. Only the vitamin B trial achieved greater than 15% randomization of underrepresented race and ethnicity participants; the range of proportions of non-Hispanic White participants was 83–95%.

**Table 2:** Demographics of randomized trial participants

	Donepezil/vitamin E (1999)	NSAIDs (1999)	Simvastatin (2002)	Vitamin B (2003)	Valproate (2003)	Huperzine A (2004)	DHA (2007)	IVIG (2008)	Resveratrol (2012)	INI (2014)	FYN (2014)
Race and ethnicity											
NH White, *n* (%)	727 (92.0)	318 (90.6)	349 (86.0)	339 (82.9)	279 (89.1)	182 (86.7)	358 (89.1)	371 (95.1)	107 (89.9)	264 (91.3)	142 (89.3)
NH Black, *n* (%)	19 (2.4)	11(3.1)	27 (6.7)	36 (8.8)	16 (5.1)	14 (6.7)	23 (5.7)	6 (1.5)	7 (5.9)	8 (2.8)	7 (4.4)
NH Asian or Pacific Islander, *n* (%)	7 (0.9)	0 (0.0)	3 (0.7)	11 (2.7)	5 (1.6)	2 (1.0)	2 (0.5)	1 (0.3)	2 (1.7)	5 (1.7)	0 (0.0)
NH American Indian or Alaskan Native, *n* (%)	3 (0.4)	3 (0.9)	1 (0.2)	0 (0.0)	1 (0.3)	0 (0.0)	3 (0.7)	0 (0.0)	2 (1.7)	0 (0.0)	1 (0.6)
Hispanic, *n* (%)	31 (3.9)	19 (5.4)	24 (5.9)	21 (5.1)	11 (3.5)	11 (5.2)	14 (3.5)	12 (3.1)	1 (0.8)	10 (3.5)	7 (4.4)
Others, *n* (%)	3 (0.4)	0 (0.0)	2 (0.5)	2 (0.5)	1 (0.3)	1 (0.5)	2 (0.5)	0 (0.0)	0 (0.0)	2 (1.0)	2 (1.3)
Age, mean (SD)	72.9 (7.3)	73.9 (7.7)	74.6 (9.3)	76.9 (8.0)	76.2 (7.9)	78.4 (8.0)	76.0 (8.7)	70.3 (9.3)	71.3 (8.1)	70.9 (7.1)	71.1 (7.7)
Sex											
Female, *n* (%)	362 (45.8)	186 (53.0)	241 (59.4)	229 (56.0)	184 (58.8)	135 (64.3)	210 (52.2)	213 (54.6)	68 (57.1)	135 (46.7)	72 (45.3)
Study partner type											
Spouse, *n* (%)	575 (72.8)	258 (73.5)	274 (67.5)	255 (62.3)	214 (68.4)	130 (61.9)	272 (67.7)	NA	93 (78.2)	248 (85.8)	130 (81.8)
Adult child, *n* (%)	113 (14.3)	71 (20.2)	102 (25.1)	121 (29.6)	74 (23.6)	67 (31.9)	104 (25.9)	NA	17 (14.3)	23 (8.0)	18 (11.3)
Others, *n* (%)	102 (12.9)	22 (6.3)	30 (7.4)	33 (8.1)	25 (8.0)	13 (6.2)	26 (6.5)	NA	9 (7.6)	18 (6.2)	11 (6.9)

The DHA trial does not indicate the age of participants above the age of 90. We report the mean (SD) age presented in the original manuscript [18]

*NA* not available

## Discussion

The last two decades have seen changes in AD clinical trial methods and criteria but few drug approvals [10, 27]. This study examined whether AD trial recruitment outcomes have changed over time within a relatively stable network of academic trial sites. We did not observe statistically significant changes over time. Instead, we observed an apparent effect of competing trials, whereby the slowest accrual was observed when most trials were simultaneously ongoing. No study achieved an overall accrual rate greater than one subject per site per month. Within trials, we observed the highest recruitment rates early in studies.

The challenges associated with study recruitment are increasingly acknowledged for delaying AD research [28–30]. These challenges may have been recognized by investigators designing these AD trials; the planned number of participants to be enrolled per site appeared to decrease over time, with two out of the last three trials conducted by the network in the reporting period having two of the lowest planned enrollments per site. This interpretation is complicated by the fact that the largest overall study was performed earliest in the assessment period, perhaps requiring greater site recruitment by the trial network, while the smallest trials were performed latest in the assessment period. These later trials’ planned enrollments may have been a product of study leaders’ desire to involve as many member sites as possible, rather than adjusted recruitment expectations, as well as other unique aspects related to the trial protocols. For example, the resveratrol trial required all participants to undergo lumbar puncture.

The accrual patterns illustrated in Fig. 1 suggest that trials occurring simultaneously are at greatest risk for delayed accrual. The vitamin B and simvastatin trials accrued at rates similar to other trials, but as the valproate and huperzine A trials were launched, each demonstrated a progressively slower accrual pattern. In striking contrast, the DHA trial, for which there were no competing trials conducted by the ADCS at the time of recruitment, was the fastest accruing study (overall 0.88 participants/site/month). This observation mirrors other fields. For example, among 787 National Cancer Institute Cooperative Group trials, studies beginning recruitment during times of higher competition (median of 4.4 competing trials per 10,000 eligible patients/year) had lower accrual rates than those conducted during the lower competition (median of 2.9 competing trials per 10,000 eligible patients per year) [31]. Similar trends of competition have also been reported in trials of stroke [32], brain tumor [33], and recent COVID-19 trials [34]. These observations may place renewed emphasis on the practice of site feasibility surveys and in particular assessing the number of competing trials that a site may have underway. While the DHA trial accrued quickly, the IVIG trial did not, despite a lack of competition from other ADCS trials. This highlights other differences that affect recruitment. The DHA trial incorporated wide eligibility criteria with few medical exclusions [18], compared to the IVIG trial [19]. DHA also was an oral supplement with a modest safety profile, compared to IVIG, which required monthly infusion visits and greater potential risks to participants.

Site performance was notably variable within trials. The range of participants enrolled per site was 0–3 at the lower extreme and 15–44 at the upper end. Furthermore, after adjusting to account for the number of active recruiting sites at a given time, we found that recruitment rates peaked early in most of the included trials (Fig. 1b). This may suggest that a small number of early launching sites are equipped and ready to enroll efficiently, while later initiating sites may accrue more slowly. A previous review of 77 human immunodeficiency virus infection trials found that early enrollment of patients, particularly in the first months, was a significant predictor of faster trial completion [35]. These early initiating sites and their success may be key to overall trial recruitment performance.

The inclusion of diverse populations is critical to ensure that the results of a study can be generalized to the larger population of people living with AD [36]. The NIH Revitalization Act of 1993 established guidelines for the inclusion of women and individuals from minority races and ethnicities in clinical research [37]. Similarly, the 2011 National Alzheimer’s Project Act recognized the racial and ethnic disparities in AD research and emphasized the federal government’s commitment to increasing enrollment of underrepresented racial and ethnic groups [38]. Despite these milestones, we observed no improvements over time in the racial or ethnic diversity of these trial populations. This result is consistent with a previous systematic review of 101 AD trials of candidate disease-modifying therapies, which showed no improvement (or worsening) in enrollment of participants from minoritized racial and ethnic groups over time between 2001 to 2019 [39]. Nearly 25% of older Americans are from a racial or ethnic minority group [40] and certain groups may be overburdened by AD [41]. Especially late-stage trials should aim to recruit representative samples of these populations but rarely do [42, 43]. In addition to the challenges of recruiting participants from underrepresented groups, participants from these groups may be more likely to be excluded based on eligibility criteria [42, 44]. The data available to us did not permit exploration of chronological differences in this potential contributor to trial representativeness. Contrary to race and ethnicity, the representation of participant sex was broadly comparable with the general MCI and AD populations. Moderate and mild-to-moderate AD trials consistently enrolled a preponderance of females, who are disproportionately affected by AD dementia [45]. Males, however, have shown a higher risk of MCI [46, 47] and made up the majority of participants in trials enrolling this diagnostic population.

### Limitations

This study had limitations. We are aware of one trial conducted by the ADCS for which data were not available [48]. This study overlapped with other trials included here, but had a total sample size of only 49 participants, recruited to only 10 sites, and had a long accrual [48]. Although the included trials were all conducted by the ADCS, there were some differences in the study design features. Factors such as trial duration, visit lengths and frequency, and treatment risks can impact participant recruitment [49] but were not included in our analyses. Lack of adjustment for these factors could have masked the effects of time. Data related to other trials (e.g., industry-sponsored trials) conducted by sites in the ADCS network during this period were not available. It is unknown whether these trials would have demonstrated differential trends over time or could have impacted the observed recruitment rates. Only one trial enrolled MCI participants. Having more MCI trials could potentially have shed light on time effects in this category of trials or differences in effects of time across diagnostic categories. Unmeasured confounds related to time may have affected the observed results. For instance, changes in patient population size (prevalence of AD at a point in time), public awareness of each trial, recruitment methods (including incentives to participate), on-site coordinators/staff, and time-related changes in the frequency of diagnosis could have masked time effects. Similarly, we cannot rule out that such confounds could have produced or altered the observed effect of competing trials.

## Conclusions

Recruitment to AD trials has been consistently challenging over the last two decades. In this analysis of studies conducted by a single trial network, no trial achieved a recruitment rate greater than one subject per site per month. Though no apparent effects of time were observed, recruitment rates appeared lower in trials that overlapped in time. Investigators may need to adjust expectations and consider the number of other currently recruiting trials when estimating accrual rates. Future analyses should examine additional predictors of recruitment rates.

## Supplementary Information

**Additional file 1: Table S1.** Key inclusion and exclusion criteria.

**Additional file 2: Table S2.** List of excluded medications.

## Data Availability

The datasets analyzed during the current study were made available through the University of California, San Diego ADCS Legacy database and through the Alzheimer’s Therapeutic Research Institute at the University of Southern California. The datasets supporting the conclusions of this article are included within the article and its additional files.

## Abbreviations

AD: Alzheimer’s disease
ADCS: Alzheimer’s Disease Cooperative Study
CI: Confidence interval
DHA: Docosahexaenoic acid
FYN: FYN kinase inhibitor
INI: Intranasally administered insulin
IVIG: Intravenous immunoglobulin
MCI: Mild cognitive impairment
MMSE: Mini-Mental State Examination
NSAIDs: Nonsteroidal anti-inflammatory drugs
