# Supplementary material for: Recruitment across two decades of NIH-funded Alzheimer’s disease clinical trials
Source: Alzheimers Res Ther. 2023 Feb 2;15:28. doi: 10.1186/s13195-023-01177-x (PMC9893207; doi:10.1186/s13195-023-01177-x)
Supplement: Supplementary file 1 — Additional file 1: Table S1. Key inclusion and exclusion criteria. [file 13195_2023_1177_MOESM1_ESM.docx]

| Supplemental Table 1: Key inclusion and exclusion criteria | | | | | | | | | | | |
| --- | --- | --- | --- | --- | --- | --- | --- | --- | --- | --- | --- |
|  | **Donepezil/ vitamin E (1999)** | **NSAIDs (1999)** | **Simvastatin (2002)** | **Vitamin B**  **(2003)** | **Valproate**  **(2003)** | **Huperzine A (2004)** | **DHA**  **(2007)** | **IVIG**  **(2008)** | **Resveratrol (2012)** | **INI**  **(2014)** | **FYN**  **(2014)** |
| INCLUSION | | | | | | | | | | | |
| Age criteria | 55-90 | ≥ 50 | ≥ 50 | ≥ 55 | ≥ 55 | ≥ 55 | ≥ 50 | 50-89 | ≥ 50 | 55-85 | 55-85 |
| MMSE criteria | 24-30 | 13-26 | 12-26 | 14-26 | 12-20 | 10-24 | 14-26 | 16-26 | 14-26 | ≥ 20 | 18-26 |
| CDR | Memory box score must be at least 0.5 | NA | NA | NA | NA | NA | NA | NA | NA | 0.5-1 | NA |
| Modified Hachinski Score | ≤4 | ≤4 | ≤4 | ≤4 | NA | ≤4 | ≤3 | ≤4 | ≤4 | ≤4 | ≤4 |
| Study partner | Required (must have an average of 10 hours per week or more contact with subject) | Required | Required | Required | Required (must have in-person contact with the participant > 2 days/week) | Required | Required (must have direct contact with the participant > 2 days/week) | Required | Required (must have direct contact with the participant > 2 days/week) | Required (must have direct contact with the participant > 2  days/week) | Required (must have an average of 10 hours per week or more contact with subject) |
| Language | NA | Fluent in English or Spanish | Fluent in English or Spanish | Fluent in English or Spanish | Fluent in English or Spanish | Fluent in English or Spanish | Fluent in English or Spanish | Fluent in English or Spanish | Fluent in English or Spanish | Fluent in English or Spanish | Fluent in English or Spanish |
| Years of education | NA | 6 years | 6 years | 6 years | NA | 6 years | NA | NA | 6 years | 6 years | 6 years |
| Lumbar puncture | NA | NA | NA | NA | NA | NA | CSF examined in subset | CSF examined in subset | Required | CSF examined in subset | CSF examined in subset |
| Mode of administration | Oral | Oral | Oral | Oral | Oral | Oral | Oral | IV infusion | Oral | Intranasal | Oral |
| MRI or CT | Within 12 months prior to SC without evidence of infection, infarction or other focal lesions | Since onset of memory impairment demonstrating absence of clinically significant focal lesion | Since onset of memory impairment demonstrating absence of clinically significant focal lesion | Since onset of memory impairment demonstrating absence of clinically significant focal lesion | Since onset of dementia consistent with the diagnosis of probable AD. Single lacunes in non-critical areas non-specific white matter changes that are interpreted as age-related are not grounds for exclusion | Since onset of memory impairment demonstrating absence of clinically significant focal lesion | Results consistent with the diagnosis of AD at some time after the  onset of the memory decline | Performed after symptom onset consistent with AD diagnosis. Excluded if there are evidence of 2 or more microhemorrhages, major stroke, or multiple lacunae by a recent (within 3 months prior to SC) and/or the baseline MRI | Excluded if there are contraindications to MRI | Excluded if there are contraindications to MRI (claustrophobia, craniofacial metal implants of any kind, pacemakers) | Excluded if SC/baseline MRI scan with evidence of infection, infarction, or other focal lesions or multiple lacunes or lacunes in a critical memory structure  and subjects that have any contraindications for MRI studies |
| PET scan | NA | NA | NA | NA | NA | NA | NA | PET imaging of cerebral glucose metabolism using ^18^F-FDG and cerebral amyloid deposition using ^18^F-AV-45 in subset | NA | NA | Required evidence of elevated fibrillary Aβ burden |
| EXCLUSION | | | | | | | | | | | |
| Depression or psychiatric disorder | Excluded with history of DSM-IV criteria for any major psychiatric disorder within 2 years. (Included if Hamilton Depression rating scale score is less than or equal to 12) | Excluded with history of DSM-IV criteria for any major psychiatric disorder within 2 years | Excluded if current DSM-IV criteria-based diagnosis for major psychiatric disorders | Excluded with history of DSM-IV criteria for any major psychiatric disorder within 2 years | Excluded with history of DSM-IV criteria for any major psychiatric disorder within 2 years | Excluded with history of DSM-IV criteria for any major psychiatric disorder within 2 years | Excluded with history of DSM-IV criteria for any major psychiatric disorder within 2 years | Excluded if major psychiatric disorders are untreated | Excluded with history of DSM-IV criteria for any major psychiatric disorder within 2 years | Excluded with history of DSM-IV criteria for any major psychiatric disorder within 2 years | Geriatric Depression Scale greater than 5 (a score ≥6 on this SC scale may be permissible, if the subject is examined by a site clinician and  judged not to be depressed). Excluded with history of major depression within 1 year |
| Psychotic features (i.e. agitation) | Excluded with history within 2 years | Excluded | NA | NA | Required for inclusion: Scores of ≤ 1 for items rating delusions, hallucinations, and agitation/ aggression taken from the NPI, modified to assess these features since onset of illness | NA | NA | Excluded if psychosis is untreated | NA | NA | Excluded with history within 3 months |
| Inflammatory disease | NA | Excluded | NA | NA | NA | NA | NA | NA | NA | NA | NA |
| Renal disease | NA | Excluded(creatinine>1.5) | Excluded if severe | Excluded | Excluded | NA | Excluded | Excluded | Excluded | Excluded | Excluded |
| B12 or folate deficiency | Excluded | NA | NA | Excluded | NA | NA | NA | NA | Excluded unless follow-up labs (homocysteine (HC) and methylmalonic acid (MMA)) indicate that it is not physiologically significant | NA | Excluded unless follow-up labs (homocysteine (HC) and methylmalonic acid (MMA)) indicate that it is not physiologically significant |
| Alcohol or substance abuse | Excluded with history within 2 years | Excluded with history within 2 years | Excluded (with current diagnosis) | Excluded with history within 2 years | Excluded with history within 2 years | Excluded with history within 2 years | Excluded with history within 2 years | NA | Excluded with history within 2 years | Excluded with history within 2 years | Excluded with history within 2 years |
| Neoplastic disease | NA | Excluded (skin tumors other than melanoma are not exclusionary; patients with stable prostate cancer may be included at the discretion of the project director) | Excluded | Excluded (skin tumors other than melanoma are not exclusionary; patients with stable prostate cancer may be included at the discretion of the Project Director) | Excluded (skin tumors other than melanoma are not excluded; patients with stable prostate cancer may be included at the discretion of the Project Director; women who have been treated for breast cancer and have no metastases and whose survival is expected to exceed 2 years may be considered for inclusion on a case-by-case basis in consultation with the Project Director; patients with purely localized bladder wall cancers may be included at the discretion of the Project Director) | Excluded (skin tumors other than melanoma are not exclusionary; participants with stable prostate cancer may be included at the discretion of the Project Director) | Excluded (skin tumors other than melanoma are not excluded; subjects with stable prostate cancer may be included at the discretion of the Project Director) | Subjects with malignancy are excluded with the exception of the following: adequately treated basal cell or squamous cell carcinoma of the skin, carcinoma in situ of the cervix, and stable prostate cancer not requiring treatment | Excluded (also excluded if history of cancer five years prior to SC, including breast cancer; history of skin melanoma or stable prostate cancer are not excluded) | Excluded (also excluded if history of cancer five years prior to SC, including breast cancer; history of skin melanoma or stable prostate cancer are not excluded) | Excluded if history within the last 5 years of a primary or recurrent malignant disease with the exception of non-melanoma skin cancers, resected cutaneous squamous cell carcinoma in situ, basal cell carcinoma, cervical carcinoma in situ, or in situ prostate cancer with normal prostate-specific antigen post-treatment |
| Hematologic disorder | NA | Excluded | NA | NA | Excluded | NA | Excluded | Excluded | Excluded | Excluded | Exclusion for CSF sub-study |
| Liver disease | NA | Excluded | Excluded | NA | NA | NA | NA | NA | NA | NA | NA |
| Residence in non-community dwelling or nursing facilities | NA | Excluded | NA | NA | Excluded | Excluded | Excluded | Excluded | Excluded | Excluded | Excluded |
| History of stroke | NA | Excluded | Excluded | Excluded | Excluded | Excluded | Excluded | Excluded | Excluded | Excluded | NA |
| Others | Any significant systemic illness that could lead to difficulty complying with the protocol | Hypersensitivity to aspirin or NSAIDs;  Active peptic ulcer disease within 5 years;  Poorly controlled hypertension;  Congestive heart failure | Conditions requiring lipid lowering drugs; LDL-cholesterol below 80 mg/dL; Other indication for the need to treat with lipid lowering drugs; Current evidence or history in the past 2 years of seizures, head injury with loss of consciousness and or immediate confusion after injury | Current evidence or history in the past 2 years of epilepsy, focal brain lesion, head injury with loss of consciousness and/or immediate confusion after the injury | Presence or previous history of agitation or psychosis requiring active psychotropic medication since the illness began; Current evidence or history in past two years of, focal brain lesion, head injury with loss of consciousness;  Evidence of any significant clinical disorder or laboratory finding that renders the participant unsuitable for receiving an investigational new drug; Clinical contraindication to the use of valproate; History of seizure within past 5 years prior to SC; Platelet count < 100,000/mm 3; International Normalized Ratio (INR) > 1.2 or Partial Thromboplastin Time (PTT) >40 seconds; Total NPI score for previous 4 weeks > 7 at SC, and for the period  between SC and Baseline | History of ulcer disease within 1 year;  Clinically significant cardiac arrhythmia;  Resting pulse less than 50;  Current evidence or history in the past 2 years of epilepsy, focal brain lesion, head injury with loss of consciousness and/or immediate confusion after the injury | Current evidence or history in past two years of epilepsy, seizure, focal brain lesion, head injury with loss of consciousness;  Evidence of any significant clinical disorder or laboratory finding that renders the participant unsuitable for receiving an investigational new drug | Clinically significant congestive heart failure;  History of unstable angina or myocardial infarction within the 12 months prior to SC; Uncontrolled hypertension;  History of thrombosis; within 12 months  Known history of procoagulant abnormalities;  Intracerebral hemorrhage within 5 years; Uncontrolled seizure disorder as defined by two or more breakthrough seizures per year despite adequate antiepileptic drug (AED) treatment;  Active autoimmune or neuro-immunologic disorder;  Poorly controlled diabetes;  Known history of or positive serology at SC for one or more of the following: hepatitis B surface antigen (HBsAg), hepatitis C virus antibody, or human immunodeficiency virus type 1/2 antibody;  Immunoglobulin A (IgA) deficiency (<7 mg/dL);  Known history of allergic reaction to albumin;  Active migraines or frequent headaches (3 or more times per week) within one year prior to SC | Diabetes; Probable AD with Down syndrome;  Current evidence or history in past two years of epilepsy, focal brain lesion, head injury with loss of consciousness;  Evidence of any significant clinical disorder or laboratory finding that renders the participant unsuitable for receiving an investigational drug;  History of seizure within past five years;  Contraindications to LP: prior lumbosacral spine surgery, severe degenerative joint disease or deformity of the spine, platelets < 100,000, use of Coumadin/warfarin, or history of a bleeding disorder;  Treated insulin dependent diabetes mellitus (Type I diabetes) and treated non-insulin dependent diabetes mellitus (Type II diabetes) | Probable AD with Down syndrome;  Current evidence or history in past two years of epilepsy, focal brain lesion, head injury with loss of consciousness;  Diabetes (type I or type II) requiring pharmacologic treatment (including both insulin dependent and non-insulin dependent diabetes mellitus;  Evidence of any significant clinical disorder or laboratory finding that renders the participant unsuitable for receiving an investigational drug;  History of seizure within past five years;  Contraindications to LP: prior lumbosacral spine surgery, severe degenerative joint disease or deformity of the spine, platelets | Clinically significant or unstable medical condition;  Exposure to radiation exceeding limit set forth in the US;  Neutropenia; Thrombocytopenia; History of interstitial lung disease;  Neutropenia defined as absolute neutrophils count of <1,800/microliter  Thrombocytopenia defined as platelet count <120x103/microliter;  Clinically significant abnormalities in SC laboratories |
| Note: “SC” = screening | | | | | | | | | | | |
