# Supplementary material for: Recruitment across two decades of NIH-funded Alzheimer’s disease clinical trials
Source: Alzheimers Res Ther. 2023 Feb 2;15:28. doi: 10.1186/s13195-023-01177-x (PMC9893207; doi:10.1186/s13195-023-01177-x)
Supplement: Supplementary file 2 — Additional file 2: Table S2. List of excluded medications. [file 13195_2023_1177_MOESM2_ESM.docx]

| Supplemental Table 2: List of excluded medications | | | | | | | | | | | |
| --- | --- | --- | --- | --- | --- | --- | --- | --- | --- | --- | --- |
|  | **Donepezil/ vitamin (1999)** | **NSAIDs (1999)** | **Simvastatin**  **(2002)** | **Vitamin B (2003)** | **Valproate (2003)** | **Huperzine A (2004)** | **DHA**  **(2007)** | **IVIG**  **(2008)** | **Resveratrol (2012)** | **INI**  **(2014)** | **FYN**  **(2014)** |
| Anti-Parkinsonian medication | Within 2 months prior to SC | Within 2 months | NA | Within 4 weeks of SC | Within 2 months of SC | Within 2 months of SC | Excluded | NA | Excluded | Excluded | Within 4 weeks of SC |
| Neuroleptic or narcotic analgesics | Within 4 weeks prior to SC | Within 2 months | Excluded | Excluded if regular use within 4 weeks or SC | Excluded if regular use within 3 months of SC | Within 4 weeks of SC | Narcotic analgesics are excluded. Some neuroleptics may be permitted at discretion of the Project Director | Short-term use (7 days or less) of narcotic analgesics  on an as needed (prn) basis will be permitted | Excluded if regularly used | Excluded | Within 4 weeks of SC |
| Benzodiazepine or barbiturates | Within 4 weeks prior to SC | NA | NA | NA | NA | NA | Excluded | Short-term use (7 days or less) of selective sedatives/benzodiazepines on an as needed (prn) basis will be permitted | NA | NA | Use of short- to medium-acting  benzodiazepines for treatment for insomnia or anxiety is permitted |
| Anxiolytics or sedative hypnotics | Within 4 weeks prior to SC | NA | NA | NA | NA | NA | NA | Excluded | NA | NA | Within 4 weeks of SC |
| Antidepressant | Within 4 weeks prior to SC | NA | Excluded (tricyclic antidepressants) | NA | Allowed if stable dose for 3 months prior to SC. Tricyclic antidepressants within 1 month prior to SC | NA | Permitted other than tricyclics and MAO inhibitors, zolpidem, trazodone and clonazepam | Permitted if stable doses for at least 6 weeks prior to SC (excludes MAOIs and tricyclics) | NA | NA | Tricyclic antidepressants are exclusionary |
| Medication with cholinergic or anticholinergic effects | Within 4 weeks prior to SC | NA | Excluded (Drugs with central anticholinergic or antihistaminic effect) | Within 4 weeks of SC | Within 2 months of SC | Within 2 months of SC | Excluded | NA | Excluded | Excluded | Within 4 weeks of SC |
| Memantine | NA | NA | NA | NA | NA | Permitted if stable dose for 3 months | Permitted if stable dose for 4 months | Permitted if stable dose for 3 months | Permitted if stable dose for 3 months | Permitted if stable dose for 12 weeks | Permitted if stable dose for 12 weeks |
| Cholinesterase inhibitors | Excluded if used within 4 weeks | Permitted if stable dose for 3 months | Permitted if stable dose for 4 weeks | Permitted if stable dose for 3 months | Permitted if stable dose for 3 months | Excluded if used within 2 months | Permitted if stable dose for 4 months | Permitted if stable dose for 3 months | Permitted if stable dose for 3 months | Permitted if stable dose for 12 weeks | Permitted if stable dose for 12 weeks |
| Anticonvulsants | Within 2 months prior to SC | NA | NA | NA | Within 5 years prior to SC | NA | NA | NA | Excluded | Excluded | Within 4 weeks of SC |
| Systemic corticosteroids | Within 3 months prior to SC | NA | NA | NA | NA | NA | NA | NA | NA | NA | Within 4 weeks of SC |
| Vitamin supplements | Within 2 weeks prior to SC | NA | NA | Use of B12 injections within 3 months of SC;  use of >250μg B12 daily within 3 months of SC;  use of >400μg folic acid daily within 3 months of SC;  use of >25mg B6 daily within 3 months of SC | Vitamin E > 2,100 IU/d within 1 month prior to SC | Use of vitamin E is allowed if the dose has been stable for 3 months prior to SC | DHA containing supplements within 2 months prior to SC. Other vitamins are permitted | NA | Vitamin E is permitted if stable dose 3 for 3 months prior to SC | NA | NA |
| Anti-inflammatory drug | NA | Regular use within 2 months | NA | NA | NA | NA | NA | Chronic use of NSAIDs within 3 months prior to SC | NA | NA | NA |
| Anticoagulants | NA | Within 2 months | NA | NA | Warfarin is permitted when approved by Project Director | NA | Stable use of Coumadin is permitted | NA | Coumadin or warfarin are not permitted | Warfarin (Coumadin) and dabigatran (Pradaxa) are not permitted | Exclusionary for CSF sub-study |
| Immunomodulatory drugs | NA | Within 3 months | Erythromycin and clarithromycin (immunosuppressives Excluded) | NA | NA | NA | NA | Immunosuppressive drugs; Immunomodulating therapies within 3 months prior to SC | NA | NA | NA |
| Estrogen | NA | Permitted if stable dose for 3 months | NA | Permitted if stable dose foe 3 months prior to SC | NA | Permitted if stable dose for 3 months prior to SC | NA | NA | Permitted if stable dose for 3 months prior to SC | NA | NA |
| Investigational drugs | Within 40 days or 5 half-lives | Within 2 months | Within 2 months | Within 2 months | Within 2 months | Within 2 months | Within 2 months | Within 3 months | Within 2 months | Within 2 months | Within 1 month |
| Others | Centrally active beta-blockers, methyldopa clonidine within 4 weeks prior to SC are not permitted | Beta-adrenergic or alpha-adrenergic receptor antagonist or agonist drugs with potential  central nervous system effects within 2 months are not permitted | Lipid lowering agents; Cyclosporin Itraconazole, ketoconazole (anti-fungal drugs); HIV protease inhibitors;  Nefazodone, verapamil;  High dose niacin;  Agents that could interact with CYP 3A4 are not permitted | Use of drugs which may influence homocysteine levels are not permitted | Psychotropics for treatment of agitation or psychosis; Zidovudine at any time;  Regular use of high doses of salicylates at SC are not permitted |  | Stable use of insulin and statins are permitted. Low dose sinemet or dopamine  agonists used no more than once a day for restless legs syndrome is permitted. | Those with IGIV treatment within 5 years prior to SC are not permitted | Use of resveratrol containing supplements (must also abstain from ingesting large quantities of resveratrol-containing foods), phenytoin, beta adrenergic or alpha adrenergic receptor  antagonist or agonist drugs with potential CNS effects, are not permitted; Drugs that are either CYP2C9 or CYP2D6 substrates and with narrow therapeutic ranges are exclusionary | Current or past use of insulin is not permitted | Mood stabilizing psychotropic agents; Carbamazepine, colchicine, cyclosporine, disopyramide, fluticasone (except as a  nasal spray which is allowable), oral steroids, quinidine, vinblastine, and vincristine are not permitted |
| Note: “SC” = screening | | | | | | | | | | | |
